# Supplementary material for: Deep Learning Approach for Differentiating Etiologies of Pediatric Retinal Hemorrhages: A Multicenter Study
Source: Int J Mol Sci. 2023 Oct 12;24(20):15105. doi: 10.3390/ijms242015105 (PMC10606803; doi:10.3390/ijms242015105)
Supplement: Supplementary file 1 [file ijms-24-15105-s001.zip › ijms-2603011-supplementary.pdf]

Supplementary Materials

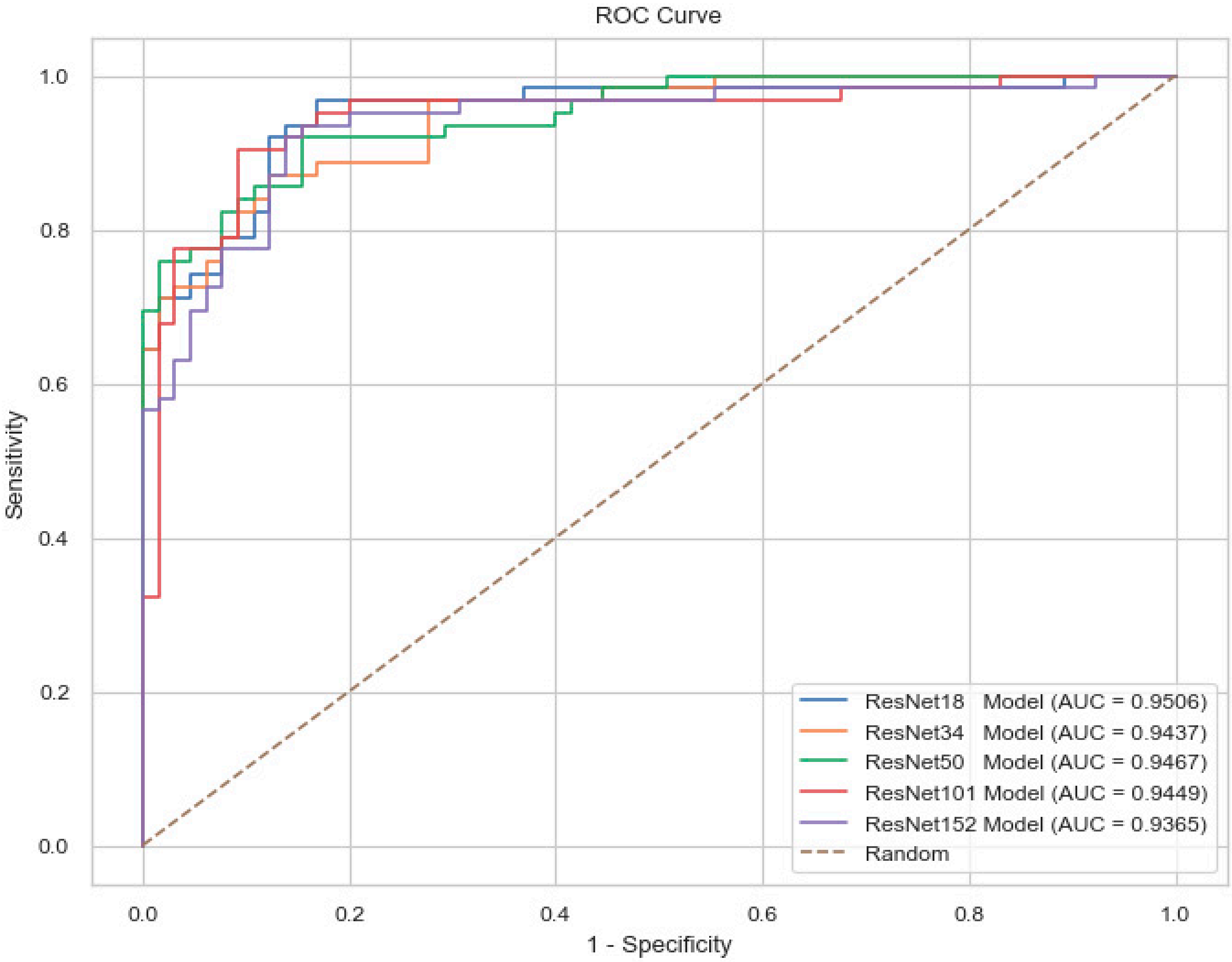

Figure S1. Under the Curve (AUC) Comparison of Various ResNet Models. This graph presents the AUC values achieved by different ResNet models, including ResNet18, ResNet34, ResNet50, ResNet101, and ResNet152, in the classification of pediatric retinal hemorrhages. The AUC scores reflect the models' performance in distinguishing between medical and trauma-induced retinal hemorrhages.

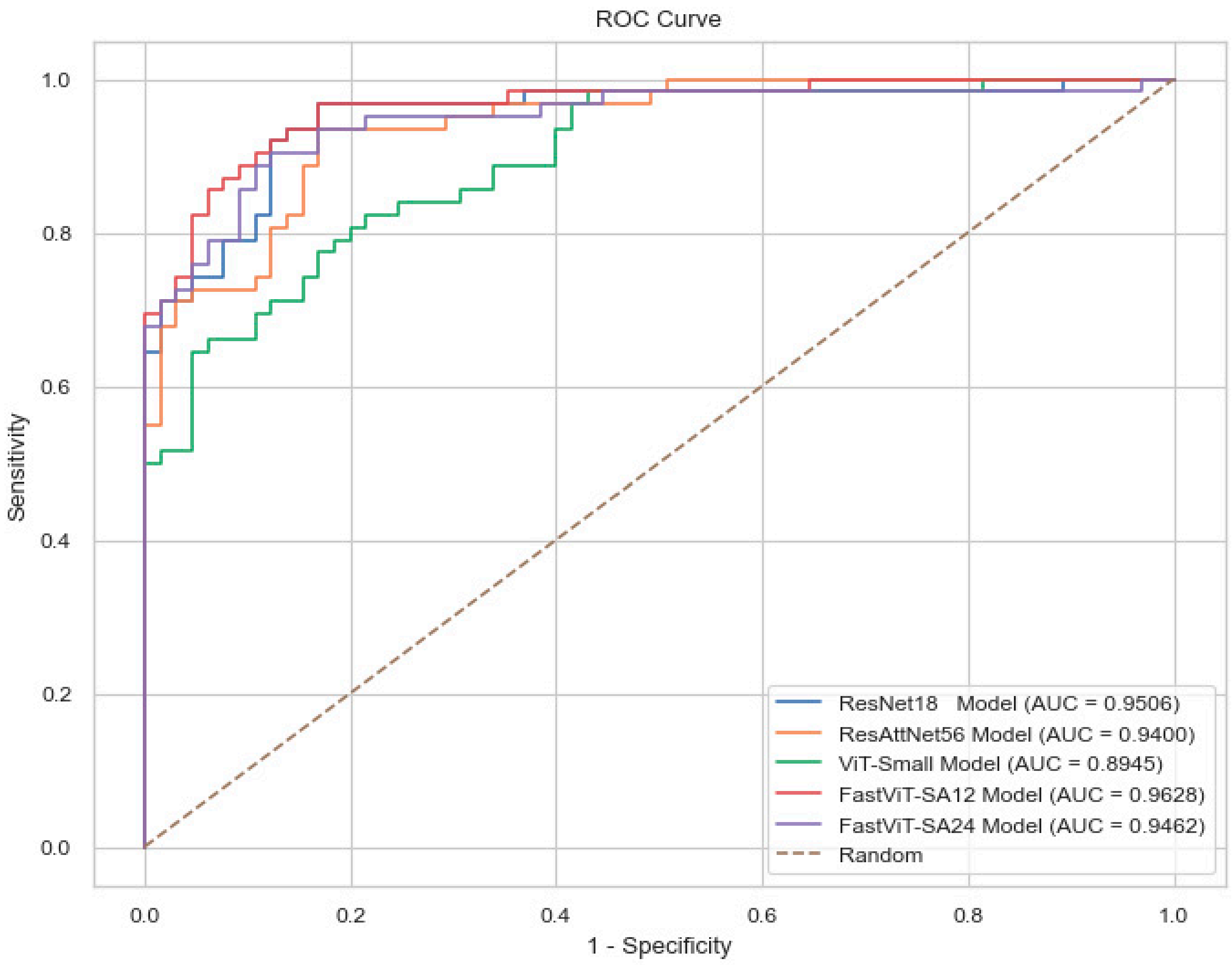

Figure S2. Under the Curve (AUC) Comparison of Top ResNet Model and Transformer Models. This graph presents the AUC values achieved by different Transformer models compared to top ResNet model, in the classification of pediatric retinal hemorrhages. The AUC scores reflect the models' performance in distinguishing between medical and trauma-induced retinal hemorrhages.

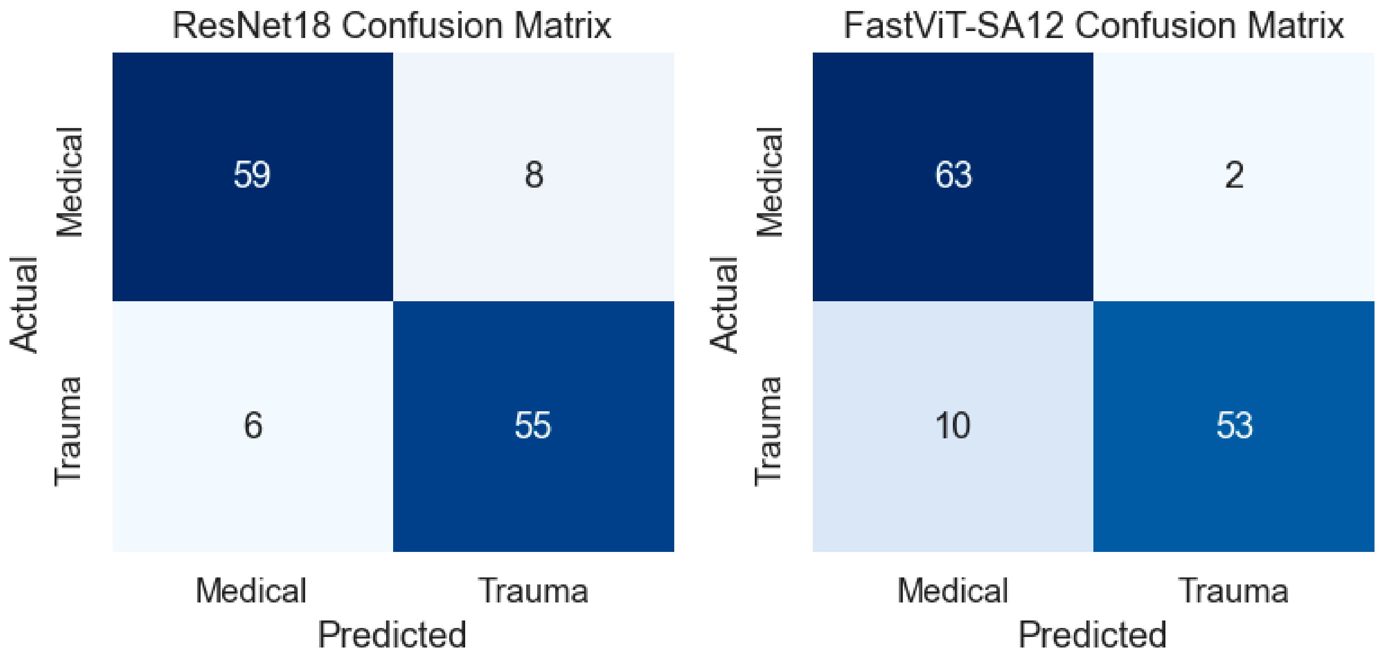

Figure S3. Confusion Matrices for Highest Sensitivity (ResNet18) and Best AUC (FastViT-SA12) Models. These matrices show the classification performance of ResNet18 (highest sensitivity) and FastViT-SA12 (highest accuracy and AUC) models in differentiating pediatric retinal hemorrhages into medical and trauma-induced categories.

Table S1. The ResNet models' misclassifications on the test subset categorized by diagnosis.

Diagnosis	ResNet18	ResNet34	ResNet50	ResNet101	ResNet152
Total misclassifications	14	17	16	13	15
Medical misclassified as trauma	8	10	9	7	8
Retinal Vascular Disease	2	2	3	1	2
Leukemia	4	5	4	4	4
Papilledema	0	0	0	0	0
Coagulopathy	2	3	2	2	2
Trauma misclassified as medical	6	7	7	6	7
Accidental Trauma	2	2	2	2	2
Birth Trauma	0	0	0	0	0
Abusive Head Trauma	4	5	5	4	5

Table S2. The ResNet18 and Transformer models' misclassifications on the test subset categorized by diagnosis.

Diagnosis	ResNet18	ResAttNet56	ViT-Small	FastViT-SA12	FastViT-SA24
Total misclassifications	14	16	26	14	15
Medical misclassified as trauma	8	11	12	2	8
Retinal Vascular Disease	2	1	3	0	2
Leukemia	4	7	7	2	5
Papilledema	0	0	0	0	0
Coagulopathy	2	3	2	0	1
Trauma misclassified as medical	6	5	14	10	7
Accidental Trauma	2	3	3	3	2
Birth Trauma	0	0	0	0	0
Abusive Head Trauma	4	2	11	7	5
